# *Vibrio parahaemolyticus* O3:K6 Epidemic Diarrhea, Chile, 2005

**DOI:** 10.3201/eid1304.06-1152

**Published:** 2007-04

**Authors:** Felipe C. Cabello, Romilio Espejo, Maria Cristina Hernandez, Maria Luisa Rioseco, Juanita Ulloa, Jose Antonio Vergara

**Affiliations:** * New York Medical College, Valhalla, New York, USA; † Universidad de Chile, Santiago, Chile; ‡ Secretaria Regional Ministerial de Salud Region X, Puerto Montt, Chile; § Hospital Regional de Puerto Montt, Puerto Montt, Chile

**Keywords:** Vibrio parahaemolyticus, climate change, epidemic diarrhea, seafood, letter

**To the Editor:** Outbreaks of diarrhea and gastroenteritis caused by *Vibrio parahaemolyticus* have been recently reported in many countries and regions where this pathogen was previously unknown (*1*,*2*). In mid-January 2005 (Figure), the number of cases of acute diarrhea produced by *V*. *parahaemolyticus* dramatically increased in Puerto Montt (41°41′S), a major city in Region X of Chile. The epidemic subsequently peaked in February and then declined with isolated cases in March and April. A total of 3,725 cases of acute diarrhea were detected during the summer months of January–April, 2005 throughout Region X (39°15′S–44°4′S). This epidemic rapidly spread to other urban areas in Region X and to the rest of Chile because Region X is the source of ≈75% of the seafood consumed in Chile. By the end of March 2005, the total number of cases in Chile was 10,783, making this the largest documented occurrence of *V*. *parahaemolyticus* diarrhea in the world.

**Figure:**
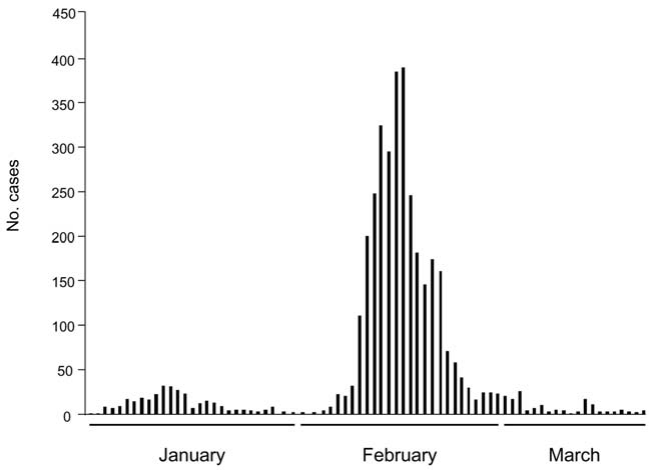
No. cases of acute diarrhea in Region X, Chile, January 4, 2005–March 21, 2005.

Analysis of a questionnaire prepared by the health authority of Region X and completed by 341 patients during January 2005 indicated that all patients had clinical signs compatible with acute diarrhea caused by *V*. *parahaemolyticus* (*3*,*4*). Stool samples of 60 patients with acute diarrhea were analyzed by standard procedures (*5*,*6*). Serotyping confirmed that all *V*. *parahaemolyticus* isolates were O3:K6 (*5*,*6*), did not produce urease, and showed the Kanagawa phenomenon (virulence-associated hemolysis) (*5**8*). PCR analysis indicated that the genome of these isolates contained *tdh*, *tlh*, and *toxRS*/new open reading frame 8 DNA sequences and lacked *trh* sequences (*1*,*7*), which are consistent with molecular characteristics of the pandemic clone O3:K6 (*1*,*5*–*8*). Pulsed-field gel electrophoresis confirmed that *V*. *parahaemolyticus* strains isolated from patients throughout the epidemic corresponded to pandemic clone O3:K6, as did the strains isolated in Chile from 2 smaller outbreaks in 1998 and 2004 (*1*,*7*). Strains of this clone also constituted the only pathogenic strain of *V*. *parahaemolyticus* detected in mussels and the only pathogenic strain that has persisted in shellfish throughout this period (*1*,*7*). The most common vectors in this outbreak were clams and mussels, not oysters, which reflect the pattern of consumption of shellfish in Chile during the summer (*1*,*7*,*9*).

This epidemic in 2005 points to the potential of *V*. *parahaemolyticus* O3:K6 to affect many susceptible persons if preventive measures are not taken and enforced quickly (*6*–*9*). Temperature and salinity have been reported as factors that influence concentrations of *V*. *parahaemolyticus* in the oceans (*2*,*5*,*7*,*8*). During summer 2005, seawater temperatures ≈19°C were recorded in several places along the coast of Region X where shellfish are collected (*7*). These temperatures were almost 3°C above 16°C, which is the average seawater temperature for January and February measured at the official weather station in Region X (http://www.shoa.cl/cendoc-jsp/index.jsp). Many of these areas with high seawater temperatures also have a wide tidal range, and shellfish in these locations are exposed to solar radiation in intertidal dry beds at ebb and low tides and can reach temperatures of 30°C. Elevated seawater temperatures and intertidal exposure to solar radiation can increase the concentration of *V*. *parahaemolyticus* in shellfish (and in the ocean), thereby increasing the risk for human infection after consumption.

Spread of *V*. *parahaemolyticus* toward the boreal and austral latitudes, as demonstrated by the course of this epidemic and the recent Alaskan outbreak, might be the result of climatic changes; a warming trend in seawater was noted in both events (*2*,*7*). Expansion of the *V*. *parahaemolyticus* O3:K6 pandemic clone may have also been facilitated by expansion of international trade because bacteria could have been transported to Chile by ballast water from the Northern Hemisphere (*1*,*4*,*6*). As in previous outbreaks, shellfish responsible for this epidemic were harvested near international shipping lanes (*1*,*3*,*4*,*6*). The appearance of *V*. *parahaemolyticus* O3:K6 in Chile has thus converted the expansion of this strain into a real pandemic because this vibrio is now present in 5 continents. The persistence of *V*. *parahaemolyticus* in Region X might also have been encouraged by an expansion of finfish and shellfish aquaculture in that area. As in other parts of the world, expansion of these food industries could provide physical and nutritional substrates for vibrios to persist and propagate when growth is triggered by increases in temperature of seawater (*1*,*2*,*7*).

Emergence of *V*. *parahaemolyticus* in Region X has also coincided with expansion of harmful algal blooms in the same area. These blooms are triggered by increases in seawater temperature and degradation of the coastal environment (*9*,*10*). A connection has been established between algal blooms and the presence of *V*. *cholerae* and cholera epidemics in the Gulf of Bengal and off the coast of Peru at the start of the Latin America epidemic (*10*). Further research is necessary to ascertain whether persistence of *V*. *parahaemolyticus* and epidemics are related to algal blooms in this region of Chile.

## References

[1] Gonzalez–Escalona N, Cachicas V, Acevedo C, Rioseco ML, Vergara JA, Cabello F, *Vibrio parahaemolyticus* diarrhea, Chile, 1998 and 2004. Emerg Infect Dis. 2005;11:129–31. 15705337 10.3201/eid1101.040762 PMC3294363

[2] McLaughlin JB, DePaola A, Bopp CA, Martinek KA, Napolilli NP, Allison CG, Outbreak of *Vibrio parahaemolyticus* gastroenteritis associated with Alaskan oysters. N Engl J Med. 2005;353:1463–70. 10.1056/NEJMoa051594 16207848

[3] Morris JG Jr. Cholera and other types of vibriosis: a story of human pandemics and oysters on the half shell. Clin Infect Dis. 2003;37:272–80. 10.1086/375600 12856219

[4] Potasman I, Paz A, Odeh M. Infectious outbreaks associated with bivalve shellfish consumption: a worldwide perspective. Clin Infect Dis. 2002;35:921–4. 10.1086/342330 12355378

[5] DePaola A, Kaysner CA, Bowers J, Cook DW. Environmental investigations of *Vibrio parahaemolyticus* in oysters after outbreaks in Washington, Texas, and New York (1997 and 1998). Appl Environ Microbiol. 2000;66:4649–54. 10.1128/AEM.66.11.4649-4654.2000 11055906 PMC92362

[6] Daniels NA, Ray B, Easton A, Marano N, Kahn E, McShan AL II, Emergence of a new *Vibrio parahaemolyticus* serotype in raw oysters. A prevention quandary. JAMA. 2000;284:1541–5. 10.1001/jama.284.12.1541 11000648

[7] Fuenzalida L, Hernandez C, Toro J, Rioseco ML, Romero J, Espejo TR. *Vibrio parahaemolyticus* in shellfish and clinical samples during two large epidemics of diarrhoea in southern Chile. Environ Microbiol. 2006;8:675–83. 10.1111/j.1462-2920.2005.00946.x 16584479

[8] Islam MS, Tasmin R, Khan SI, Bakht HB, Mahmood ZH, Rahman MZ, Pandemic strains of O3:K6 *Vibrio parahaemolyticus* in the aquatic environment of Bangladesh. Can J Microbiol. 2004;50:827–34. 10.1139/w04-072 15644897

[9] Hernandez C, Ulloa J, Vergara JA, Espejo R, Cabello F. *Vibrio parahaemolyticus* infections and algal intoxications as emergent public health problems in Chile. Rev Med Chil. 2005;133:1081–8. 16311702 10.4067/s0034-98872005000900013

[10] Colwell RR. Infectious disease and environment: cholera as a paradigm for waterborne disease. Int Microbiol. 2004;7:285–9. 15666250

